# Engendering High Energy Density LiFePO_4_ Electrodes with Morphological and Compositional Tuning

**DOI:** 10.3390/nano13111771

**Published:** 2023-05-31

**Authors:** Aleksei V. Kubarkov, Alexander V. Babkin, Oleg A. Drozhzhin, Keith J. Stevenson, Evgeny V. Antipov, Vladimir G. Sergeyev

**Affiliations:** 1 Department of Chemistry, Lomonosov Moscow State University, Leninskie Gory 1-3, 119991 Moscow, Russia; a.v.babkin93@yandex.ru (A.V.B.); drozhzhin@elch.chem.msu.ru (O.A.D.); kjsaustin@msn.com (K.J.S.); antipov@icr.chem.msu.ru (E.V.A.); 2 Center for Energy Science and Technology, Skolkovo Institute of Science and Technology, Bolshoy Boulevard 30 bld. 1, 121205 Moscow, Russia

**Keywords:** Li-ion battery, LFP, carbon nanotube, particle morphology, current collector, carbon coating, electrode composition, areal capacity, adhesion, peel resistance

## Abstract

Improving the energy density of Li-ion batteries is critical to meet the requirements of electric vehicles and energy storage systems. In this work, LiFePO_4_ active material was combined with single-walled carbon nanotubes as the conductive additive to develop high-energy-density cathodes for rechargeable Li-ion batteries. The effect of the morphology of the active material particles on the cathodes’ electrochemical characteristics was investigated. Although providing higher packing density of electrodes, spherical LiFePO_4_ microparticles had poorer contact with an aluminum current collector and showed lower rate capability than plate-shaped LiFePO_4_ nanoparticles. A carbon-coated current collector helped enhance the interfacial contact with spherical LiFePO_4_ particles and was instrumental in combining high electrode packing density (1.8 g cm^−3^) with excellent rate capability (100 mAh g^−1^ at 10C). The weight percentages of carbon nanotubes and polyvinylidene fluoride binder in the electrodes were optimized for electrical conductivity, rate capability, adhesion strength, and cyclic stability. The electrodes that were formulated with 0.25 wt.% of carbon nanotubes and 1.75 wt.% of the binder demonstrated the best overall performance. The optimized electrode composition was used to formulate thick free-standing electrodes with high energy and power densities, achieving the areal capacity of 5.9 mAh cm^−2^ at 1C rate.

## 1. Introduction

LiFePO_4_ (LFP) is a promising active cathode material for manufacturing high-energy-density Li-ion batteries for electrical vehicles and industrial energy storage systems [1,2]. Further development of efficient LFP batteries requires maximizing both gravimetric and volumetric energy density and specific power of cathodes [3,4]. The volumetric power density of a composite electrode is determined by its rate capability as well as the compaction density of the active material. Both of these parameters depend highly on the morphology of the active material particles, as shown in multiple studies [5,6,7,8].

LFP is a one-dimensional ionic conductor in which the charge transfer takes place on the (010) crystal facet, while Li^+^ ions diffuse along the [010] direction that is perpendicular to the (010) surface [9]. Plate-shaped nanoparticles are considered the best in terms of measured rate capability since this morphology of LFP presumably exposes the (010) facets to the electrolyte and provides the shortest diffusion distance of lithium ions in the solid LiFePO_4_ phase [7,10]. However, the compaction and therefore the tap density of such nanoplates are relatively low [8]. The highest compaction density is provided by micro-sized spherical LFP microparticles that, however, demonstrate poor rate capability [8]. The rate performance of LFP microspheres can be improved by developing a hierarchical porous microstructure that consists of nano-sized primary nanoparticles [6,11,12]. Thus, it is challenging to attain a good rate capability and tap density of the active material particles simultaneously, and further optimization of the LFP morphology is required.

Besides the active material, cathodes incorporate electrochemically inactive components such as polymer binders and electrically conductive additives, which also influence the volumetric power density. Their content should be minimized to attain a higher compaction density of the cathode. The amount of the conductive component could be reduced by replacing conventionally used carbon black (CB) with carbon nanomaterials (nanotubes, graphene) that exhibit high conductivity at lower percolation thresholds [13,14,15,16]. For example, the percolation threshold of single-walled nanotubes in lithium nickel cobalt oxide (NMC) cathodes is only 0.01 vol.% while that of CB reaches 0.7–0.9 vol.% [16]. Carbon nanotubes (CNTs) have been recently adopted as a conductive agent for LFP [17,18] and several other cathode [14] and anode [13,19] materials for Li-ion batteries. Several studies compared the performance of carbon CNTs and CB in composite electrodes. The CNT electrodes demonstrated an enhanced rate capability and cycle performance [14,17,19,20,21] as well as decreased polarization voltage [17] in comparison to electrodes made with CB. This improvement was related to the ability of nanotubes to form conductive bridges between particles of the active material [14]. The content of CNTs may affect several important characteristics of the electrode, including its conductivity and volumetric capacity [18]. Hence, the composition of CNT electrodes requires optimization.

Another promising way of increasing the power density of battery electrodes is optimizing the surface structure of a current collector. It efficiently increases the capacity of the electrodes without affecting their volume-based characteristics. The current collector can be modified with carbon coating [22] which improves the roughness and wettability of the current collector’s surface [23] and its adhesion to the active layer of the electrode [24].

In the present study, we developed high-energy-density LFP cathodes by combining different approaches such as morphology control of LFP particles, modification of a current collector, and use of a nanostructured conductive additive. The study aimed to investigate the electrochemical performance of the LFP-based composite electrodes, which contain single-walled carbon nanotubes (SWCNT) and LFP particles of the two promising morphologies (plate-shaped nanoparticles and spherical microparticles) and optimize the electrode composition to achieve the best electrochemical properties.

## 2. Materials and Methods

Plate-shaped LFP microparticles were synthesized via a solvothermal route as we described earlier [25]. Spherical LFP particle agglomerates were provided by BTR New Energy Materials (Shenzhen, China). Carbon content in the plate-shaped and spherical LFP was determined by the thermogravimetric analysis [26] as ca. 2.0 and 3.2 wt.%, respectively.

Single-walled carbon nanotubes Tuball^TM^ (SWCNT, OCSiAl, Novosibirsk, Russia, length > 5 µm, G/D ratio > 40) were purified from metallic impurities in hydrochloric acid [27] before use. Polyvinylidene fluoride (PVDF, Solef 5130, Solvay S.A., Brussels, Belgium) and *N*-methyl-2-pyrrolidone (NMP, Acros Organics, Geel, Belgium) were used as received from the manufacturer.

Aluminum foil (Al) and carbon-coated aluminum foil (Al-C) were supplied by Gelon LIB (Shandong, China). The structure of the carbon coating was characterized by Raman spectroscopy. The spectra were acquired with a DXRxi Raman Imaging Microscope (Thermo Fisher Scientific, Madison, WI, USA) using a 532 nm excitation laser (2.0 mW power). The laser beam was focused on the carbon coating, and the Raman signal was acquired in the back-scattering direction. The spectra recorded in 10 different places of the carbon coating were similar, indicating the uniform structure of the coating. The band intensity ratio *I*_D1_/*I*_G_ was 1.3 (Figure S1), indicating the disordered structure of carbon.

The required amount of SWCNT was mixed with 0.8 mL of NMP, then ultrasonicated for 10 min using a Vibra-Cell VCX 750 ultrasonic processor (Sonics Materials Inc., Newton, CT, USA), followed by stirring on a magnetic stirrer at a constant speed for 24 h. Next, 0.2 mL of pre-prepared PVDF solution (10% *w*/*v* in NMP) were added to the SWCNT dispersion; the resulting SWCNT dispersion was uniform and stable to sedimentation with no macro-phase separation after several weeks. Then the required mass of LFP was added, and the dispersion was stirred for 24 h.

The prepared electrode slurry was applied to an Al or Al-C current collector with an applicator following the doctor-blade method [28] and leaving a casting gap of 300 µm. After drying at 60 °C, the sheet was roll-pressed with a roll gap of ca. 40 µm and stamped into disk electrodes with an area of 2 cm^2^. The electrodes were weighed and dried at 110 °C in a vacuum (0.01 atm) overnight. The electrodes were formulated with different LFP:PVDF ratios and contained 95–98 wt.% of LFP, 1.75–4.75 wt.% of PVDF, and 0.05–0.5 wt.% of SWCNT. The electrodes were prepared with equal surface loadings of LFP (4.7 ± 0.3 mg cm^−2^).

The densities of the electrodes were calculated using the following equation:

(1) $$d=\frac{m_{c}-m_{f}}{A\times t},$$

where *m*_c_ and *m*_f_ are the masses of an electrode and current collector foil, respectively; *A* is the electrode area (2 cm^2^); and *t* is the coating thickness determined as the average value of 10 measurements by a digital micrometer.

Thicker free-standing electrodes were produced with higher LFP loadings (18 and 45 mg cm^−2^) by adjusting the doctor-blade gap to 600–1200 µm. After the roll-pressing, thick electrodes were carefully delaminated from the current collector surface to create free-standing electrode structures.

Scanning electron microscopy (SEM) images of the LFP particles were obtained using a JEOL JSM-6490LV microscope (JEOL, Tokyo, Japan). Cross-sections of the composite electrodes and individual spherical LFP particles were prepared by xenon plasma focused ion beam (FIB) using a Helios G4 PFIB UXe DualBeam microscope (ThermoFischer Scientific, Walthem, MA, USA).

Powder X-ray diffractograms of LFP samples were obtained using a Huber G670 Guinier Camera (CuK*α*_1_ radiation).

The electrical conductivity of the electrodes was measured by the four-probe method using a Loresta GP MCP T610 resistivity meter (Mitsubishi Chemical, Tokyo, Japan). The electrode films for the conductivity measurement were prepared from the electrode slurries that were obtained as described above. The films (10–15 µm thick) were formed on a flat non-conductive silicate glass surface by the drop-casting method and dried at 50 °C until constant weight.

Electrochemical half-cells were assembled in a LABstar glove box (mBraun, Garching, Germany) in an Ar atmosphere (*p*(O_2_) < 0.5 ppm, *p*(H_2_O) < 0.5 ppm). 1 M LiPF_6_ in the mixture of ethylene carbonate and diethyl carbonate (1:1 *v*/*v*) was used as the electrolyte. Glass fiber film (Schleicher & Schuell MicroScience, Dassel, Germany) was used as a separator. Galvanostatic experiments were carried out using an Elins P-20X8 potentiostat (Electrochemical Instruments, Chernogolovka, Russia). Galvanostatic charge/discharge experiments were carried out in the potential range of 2.0–4.1 V vs. Li/Li^+^. Specific capacities were normalized to the mass of the active material (LFP). Each electrochemical experiment was performed in 2–3 different cells in parallel under the same conditions. The relative standard deviation of the measured capacity values was below 5%.

The electrochemical impedance spectra of the cells were measured at 50% degree of discharge using an Autolab PGSTAT302N potentiostat/galvanostat (Metrohm Autolab B.V., Utrecht, The Netherlands) at open circuit potential within the frequency range of 100 kHz to 0.1 Hz (5 points per decade) and with a voltage amplitude of 5 mV.

The peeling force of the composite electrodes was measured following the standard method ASTM D1876-01 [29]. Peeling tests were carried out on electrode samples with a length of at least 3 cm at a speed of 100 mm min^−1^ using a universal testing machine EZ-LX (Shimadzu Europa, Duisburg, Germany). The detailed procedure was reported earlier [25]. The peeling forces were normalized by the width of the peeling tape (1.9 cm). The peeling strength of each electrode was calculated as the average of three peeling tests. Optical microscopy images of the current collectors were taken using an Axioskop 40 optical microscope (Carl Zeiss, Jena, Germany).

## 3. Results

### 3.1. Effect of LFP Morphology

**Figure 1:**
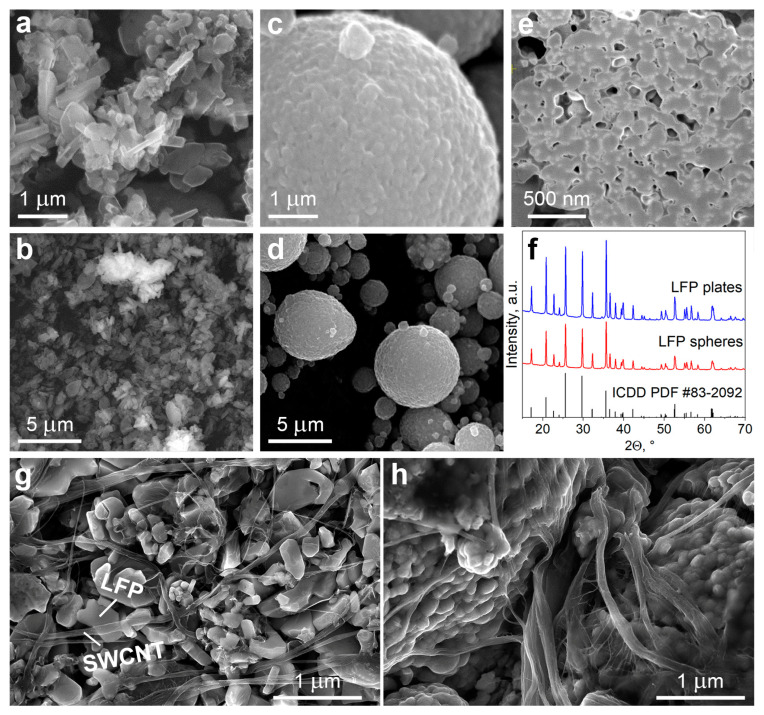
SEM micrographs of (**a**,**b**) plate-shaped and (**c**,**d**) spherical LFP particles at different magnifications; (**e**) cross-sectional SEM view of an individual spherical LFP particle; (**f**) XRD patterns of LFP particles as compared to the ICDD PDF pattern of LiFePO_4_; SEM images of the surfaces of the composite electrodes prepared with (**g**) plate-shaped and (**h**) sphere-shaped LFP particles (the electrodes contained 95 wt.% of LFP, 4.75 wt.% of PVDF, and 0.25 wt.% of SWCNT).

The morphological features of LFP particles are examined by SEM (Figure 1a–d).

As shown in SEM micrographs, plate-shaped LFP particles are nanoscale with an average width of 50–100 nm and a length of about 0.5–1 µm, while spherical LFP particles are much larger, and their diameter varies from 1 to 10 µm. Plate-shaped LFP particles have a well-defined shape and smooth edges (Figure 1a,b), while sphere-shaped particles have a rough surface (Figure 1c,d).

The large size and high roughness of spherical particles suggest that they do not have a monolithic structure and may consist of agglomerated smaller particles, which is typical for spherical LFP material [30]. For this reason, we studied the morphology of spherical LFP particles in more detail by analyzing cross-sections of particles using the focused ion beam (FIB) technique. The cross-sectional view of an individual LFP spherical particle (Figure 1e) confirms the presence of densely agglomerated smaller spherical nanoparticles with an average diameter of ca. 200 nm. The primary particles are located close to each other which results in a high tap density of this material (1.7 mg cm^−3^). There are, however, some cavities between the primary LFP particles, which can be filled by liquid electrolytes. Wetting the LFP primary particles with electrolyte enables them to fully exploit the active material capacity and fast lithium-ion intercalation/deintercalation kinetics [10]. It is also worth noting that spherical LFP microparticles are highly polydisperse (Figure 1d). The broad size distribution ensures a large contact area between the secondary LFP particles and, consequently, a high tap density since the smaller particles can fill into the cavities between the larger ones [30,31,32]. Compared to other irregular LFP morphologies, spherical LFP particles are also advantageous for achieving higher tap density of LFP cathodes [32].

Figure 1f shows the XRD patterns of the plate- and sphere-shaped LFP particles. Both types of materials demonstrate pure LiFePO_4_ phases with olivine structure (PDF #83-2092). Narrow and sharp diffraction peaks strongly indicate the crystalline LiFePO_4_ phase.

Plate- and sphere-shaped LFP particles were used to fabricate the electrodes. SEM images of the electrode surfaces are compared in Figure 1g–h. SWCNT bundles with an average diameter of ca. 10–100 nm and a length of a few microns are seen clearly in both electrodes. The electrode formulated with plate-shaped LFP contains nanotube bundles that are well-dispersed between individual LFP particles (Figure 1g). As a result, the SWCNT bundles form an interconnected network contacting most of the LFP particles. The electrode prepared with spherical LFP particles shows a less uniform distribution of SWCNT (Figure 1h). In this case, LFP particles are much larger than the nanotube bundle diameter. The nanotubes are mostly located at the surface of LFP agglomerates (Figure 1h). However, the nanotubes cannot penetrate the LFP agglomerates to form contacts with primary particles in the bulk, since the diameter of the nanotube bundles exceeds the pore size (Figure 1e).

**Figure 2:**
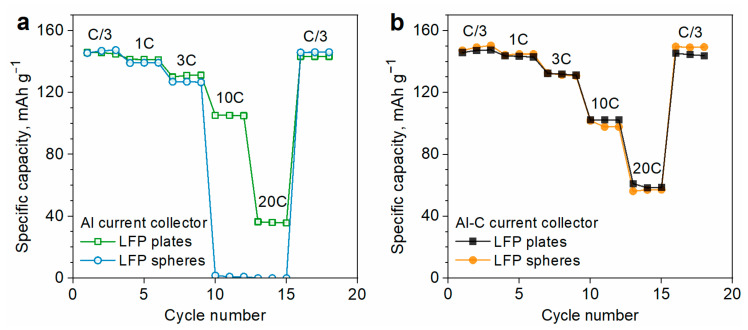
Rate capability of the composite electrodes that are prepared from the spherical and plate-shaped LFP particles on the (**a**) Al and (**b**) Al-C current collectors. The electrodes contained 95 wt.% of LFP, 4.75 wt.% of PVDF, and 0.25 wt.% of SWCNT.

Specific capacities of the composite electrodes formulated with LFP particles of different morphologies are compared in Figure 2a. The mass content of the active material (LFP), polymer binder (PVDF), and SWCNT are similar in both electrodes.

Both electrodes deliver the same capacity of 146 mAh g^−1^ at the rate of C/3 and demonstrate almost identical rate performance at the rates of C/3–3C. However, the capacitive behavior of the electrodes differed at higher charge/discharge rates (10C–20C). The electrode prepared with plate-shaped LFP demonstrates a high specific capacity of 105 mAh g^−1^ at 10C, while the one prepared with spherical LFP exhibits a much poorer rate performance (almost zero capacity at 10C) (Figure 2a). One can consider two possible explanations for this fact. First, SWCNT bundles are distributed more uniformly in the electrode prepared with plate-shaped LFP particles (Figure 1g–h), so they provide better electrical contact with the plate-shaped LFP material. Another possible reason is that the morphology of LFP particles affects the resistance of the electrical contact on the current collector. LFP particles of different shapes might contact the current collector differently, which can influence the interfacial contact resistance and thus affect the capacity behavior [24]. To examine this issue, we have prepared the electrodes on the surface of a carbon-coated aluminum current collector. Coating the current collector with carbon is a well-known approach for improving the electrical contact of the current collector with the electrode’s active particles [22,33].

**Figure 3:**
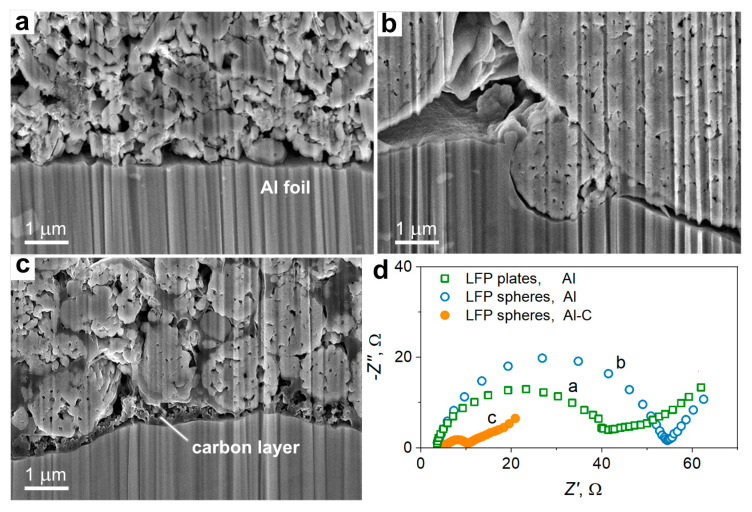
Cross-sectional SEM micrographs of the electrode/current collector interfaces for the electrode that are prepared with (**a**) plate-shaped and (**b**) sphere-shaped LFP particles on a bare Al current collector or (**c**) sphere-shaped LFP particles on an Al-C current collector; (**d**) Nyquist plots of the corresponding composite electrodes.

Figure 2b shows the rate performance of the electrodes prepared on carbon-coated aluminum foil (Al-C). Using a carbon-coated current collector significantly improves the rate capability of spherical LFP particles but has only a minor influence on the rate performance of plate-shaped LFP (Figure 2a,b). As a result, both types of LFP particles demonstrate almost identical rate capability if prepared on the Al-C foil (Figure 2b). Thus, the difference in the rate capability of LFP plates and LFP spheres depends largely on the properties of the current collector. Hence, we can assume that electrochemical performance of the electrode is primarily affected by the contact of LFP particles with the current collector. The efficiency of this contact is influenced by the morphology of LFP particles and surface structure of the current collector. In fact, sphere-shaped particles are expected to have a smaller contact area with the plain current collector as compared with plate-shaped ones, while the morphology of Al and Al-C current collectors may additionally influence this area. The wider contact area is expected to result in lower contact resistance at the electrode/current collector interface and better rate capability. To investigate the electrode/current collector interface in more detail, we have inspected the SEM images of the electrode cross-sections prepared by a focused ion beam (Figure 3a–c).

The electrode formulated with plate-shaped LFP has a smooth interface area with LFP particles directly contacting the aluminum current collector (Figure 3a). Spherical LFP particles demonstrate poorer contact with Al foil; there are gaps and cavities between the active material particles and the current collector with only limited areas of direct contact (Figure 3b). The surface of the current collector is not plane and is deformed by the larger LFP microspheres (Figure 3b) as a result of the roll-pressing procedure. Hence, sphere-shaped LFP particles have a smaller contact area with the aluminum current collector than plate-shaped ones [34]. The surface of the carbon-coated current collector (Al-C) contains a continuous 200–400 nm thick carbon layer (Figure 3c). This layer fits efficiently into the gap between the aluminum foil and the adjacent LFP particles, thus providing good contact between them (Figure 3c).

We further characterized the electrodes with impedance spectroscopy to get a better insight into the correlation between the structure of the electrode/current collector interface and the electrical contact resistance. The high-frequency intersection of the spectra is related to the resistance of the electrolyte, which is nearly the same (ca. 4 Ω) for all the studied electrodes (Figure 3d). In the medium-frequency range, the spectra contain a semicircle that represents the charge transfer resistance (*R*_ct_) [35]. The electrical resistance at the electrode/current collector interface contributes to the total *R*_ct_, and, therefore, the change of interfacial contact resistance affects the measured *R*_ct_ value [24]. The electrodes prepared on a bare Al foil demonstrate lower *R*_ct_ for plate-shaped LFP particles (ca. 35 Ω) than spherical ones (ca. 50 Ω, Figure 3d). This is consistent with the observation of a smoother contact area for the plate-shaped particles (Figure 3a,b). The type of current collector significantly impacts the value of *R*_ct_ measured for the spherical particles. The electrode prepared on the aluminum current collector (Al) has a high *R*_ct_ (ca. 50 Ω), while a much lower *R*_ct_ of 5 Ω is obtained for the electrode formulated on the Al-C current collector (Figure 3d). Hence, the analysis of impedance spectra confirms that a carbon-coated current collector provides better electrical contact with spherical LFP particles.

Figure S2 compares the galvanostatic profiles of the studied electrodes at 1C charge/discharge rates. Although the specific capacities of the electrodes are the same, the voltage hysteresis between the lithiation and delithiation plateaus varies much, depending on the morphology of LFP particles and the type of the current collector. The electrodes prepared on the bare Al foil demonstrate much lower voltage hysteresis with LFP plates (92 mV) as compared to LFP spheres (255 mV), which can be attributed to the difference in charge transfer resistances of these electrodes [36]. Using a carbon-coated current collector significantly reduces the resistance of the latter electrode (Figure 3d) and, therefore, reduces the overvoltage in the galvanostatic experiment. As a result, voltage hysteresis decreases to 156 mV (Figure S2).

In addition to high specific capacity, it is essential for the cathodes to have high packing density, because this parameter directly influences the volumetric energy density of the battery. The packing densities of the composite electrodes prepared with LFP particles of different morphologies are compared in Table 1.

Spherical LFP particles demonstrate higher tap density and provide a denser electrode structure with twice the packing density and volumetric capacity of plate-shaped particles (Table 1). As noted above, high packing densities are typical for the secondary LFP microparticles that are characterized by broad particle size distribution [30,31,32]. However, the packing density of our electrode formulated with the spherical LFP particles (1.76 g cm^−3^) is even higher than that of similar high-energy-density LFP cathodes reported earlier in the literature (1.3–1.5 g cm^−3^) [18,37,38,39]. The enhancement of packing density can be attributed to the low content of the electrically conductive component (SWCNT) and the shape of LFP particles that form a more densely packed material.

Thus, it can be concluded that spherical LFP agglomerates demonstrate high packing density but contact poorly with the traditional aluminum current collector, which results in increased interfacial resistance. A carbon-coated current collector provides better interfacial contact with spherical LFP and improved rate capability. In other words, the electrodes prepared with spherical LFP particles and an Al-C current collector are the most optimal in terms of energy density. For this reason, electrodes of this type were used for further experiments. The following stage of the work is aimed at studying the effect of SWCNT content on the electrochemical characteristics of the electrodes.

### 3.2. Effect of the SWCNT Content

**Figure 4:**
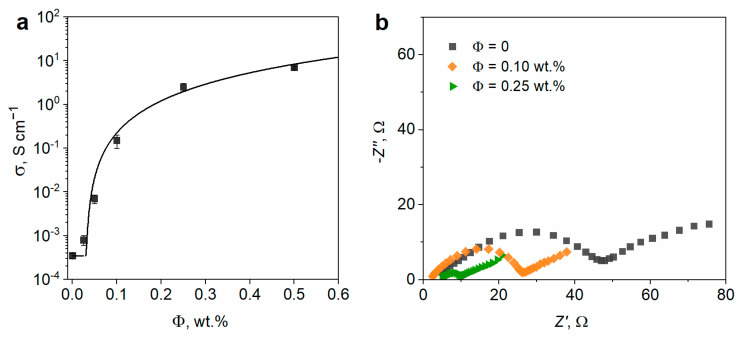
(**a**) Electrical conductivity of composite cathodes as a function of the SWCNT weight content; (**b**) Nyquist plots (50% state-of-discharge) of the composite cathodes prepared with different contents of SWCNT. The cathodes contained 95 wt.% of sphere-shaped LFP particles.

Conductivity was measured for cathode composites that were prepared with different contents of SWCNT (Figure 4a).

The electrodes formulated without SWCNT demonstrate electrical conductivity of 3.5 × 10^−4^ S cm^−1^ originating from the conductive carbon coatings on the surface of LFP microparticles. The conductivity of electrodes increases up to 0.15 S cm^−1^ with increasing the SWCNT content (Φ) up to 0.1 wt.% and reaches a plateau (~1 S cm^−1^) at higher SWCNT loadings (0.25–0.5 wt.%). The values of the electrical conductivity fit the equation:

(2) $$\sigma =\sigma_{\mathrm{LFP}/C}+\sigma_{0}{\left(\Phi -\Phi_{c}\right)}^{t},$$

with σ_LFP/C_ = 3.5 × 10^−4^ S cm^−1^ —conductivity of PVDF-LFP/C composite with no SWCNT added, Φ*_c_* = 0.03 wt.%—fitted percolation threshold of the SWCNT, σ_0_ = 35 S cm^−1^ and *t* = 1.9—fitting coefficients [40]. The estimated Φ*_c_* value is lower as compared to the earlier reported electrical percolation threshold of SWCNT (0.08 wt.%) [41] and Tuball^TM^ SWCNT (0.1–0.2 wt.%) [42] in the PVDF matrix. The electrical conductivity of the carbon coating on the surface of LFP may account for the lower percolation threshold found in our work, as it can provide additional electrical pathways by interacting with SWCNT. Moreover, within the electrode structure, carbon nanotubes are segregated at the surface of the large LFP agglomerates (Figure 1h). The formation of such segregated composites is known to significantly reduce the electrical percolation threshold in contrast to the system with randomly distributed nanotubes [43]. This can result in a lower percolation threshold of SWCNT in the electrode as compared to that in PVDF polymer. In fact, the conductivity percolation threshold of Tuball SWCNT in the NMC cathode is reported to be only 0.02–0.06 wt.% [44].

The electrochemical impedance spectra of the electrodes (Figure 4b) are in good agreement with the direct current conductivity data (Figure 4a). With an increase in the content of SWCNT, charge transfer resistance decreases, reaching the lowest value at 0.25 wt.% of SWCNT (Figure 4b).

The galvanostatic profiles of the electrodes with different SWCNT contents are compared in Figure S3. The electrodes demonstrate good reversibility at C/10 with a first-cycle CE of 97–99%, reaching 99.6–100% at the following cycles. The addition of SWCNT reduces the voltage hysteresis between the lithiation and delithiation plateaus from 140 mV (SWCNT-free electrode) to 55 mV (0.25 wt.% SWCNT), while the specific capacity is increased from 136 to 149 mAh g^−1^ (Figure S3).

**Figure 5:**
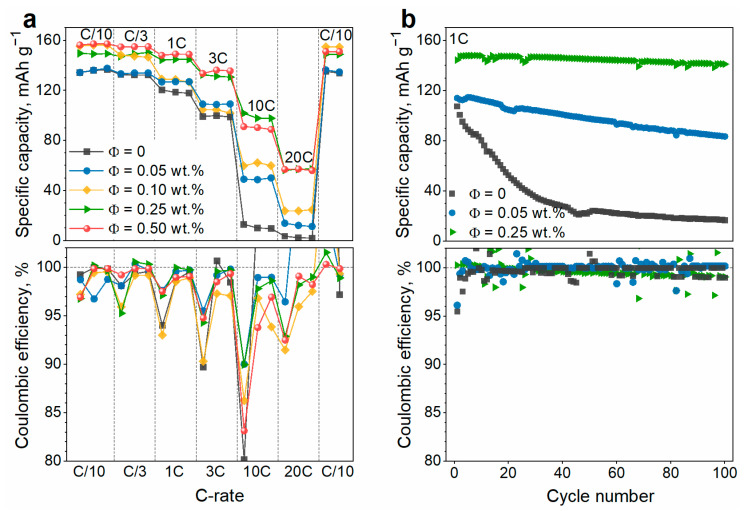
(**a**) Rate capability and (**b**) cycling performance (1C-rate) of the composite cathodes incorporating 95 wt.% of spherical LFP particles and different contents of SWCNT.

The influence of the SWCNT content on capacity characteristics of the electrodes at different charge/discharge rates is illustrated in Figure 5a. At the end of testing, two more cycles are performed at the lowest rate (C/10) to confirm that no significant capacity fading was observed during experiment and so the rate performance of the electrodes was estimated correctly.

The electrode without SWCNT demonstrates poor capacity at high charge/discharge rates of 10C and higher. Even a minor addition of SWCNT (0.05 wt.%) significantly increases the capacity at high rates (Figure 5a). Increasing the SWCNT content up to 0.25 wt.% consistently improves the rate performance of the electrode. Nevertheless, a further increase in SWCNT amount up to 0.50 wt.% does not change the capacity values (Figure 5a). The effect of SWCNT on the electrode rate capability is associated with the influence of carbon nanotubes on the conductivity of composite electrodes [16]. The correlation between the electrical conductivity and the high-rate capacity of LFP cathodes is well established in the literature [16,45]. A strong increase in both conductivity (Figure 4a) and rate capability (Figure 5a) of the electrodes is observed upon the addition of a small amount of SWCNT (0.10–0.25 wt.%). However, no further improvement of the rate performance is observed at higher SWCNT loadings (Φ*_c_* > 0.25 wt.%) (Figure 5a), since the rate of the electrochemical reaction becomes limited by other factors besides electrical conductivity, e.g., ionic resistance of the electrolyte and the solid-state diffusion rate of the lithium ions [16]. Thus, the rate performance is independent of the electrical conductivity at Φ*_c_* ≥ 0.25 wt.%.

The coulombic efficiency (CE) of the electrodes exceeds 99.7% at low charge/discharge rates (C/10–3C), with no obvious correlation between the CE values and SWCNT content (Figure 5a). At each charge/discharge rate, the electrodes demonstrate lower CE value at the first cycle as compared to the two following cycles. This is because the lower current density provides a deeper discharge of an electrode and, as a result, an increased first-cycle charge capacity at the next (higher) current density. The electrode with 0.25 wt.% SWCNT demonstrates a CE of 95–97% for the first cycles at different C-rates and 99.8–100% for the following cycles. At higher charge/discharge rates (10–20C), the polarization of the electrodes increases and the lithiation plateau approaches the upper cutoff potential (4.1 V vs. Li/Li^+^), thus affecting the CE values. The electrode without SWCNT fails to perform at 10C–20C due to the low electrical conductivity (Figure 4a). The electrodes with 0.25–0.5 wt.% of SWCNT are less polarized and demonstrate a more reversible high-rate performance (CE of 98.3–99.1% at 20C).

The capacity data shown in Figure 5a allows us to conclude that the optimal amount of SWCNT in a composite cathode is 0.25 wt.%, while further addition of SWCNT is impractical. It should be noted that we determined the optimal amount of SWCNT (0.25 wt.%) only for the electrodes formulated with a fixed amount of LFP (95 wt.%). However, the actual amount of LFP and PVDF might also influence the electrochemical performance of electrodes [46,47]. For this reason, we prepared electrodes that contain various amounts of LFP (95–98 wt.%), PVDF (1.75–5.0 wt.%), and SWCNT (0–0.5 wt.%) and examined their electrochemical performance (Figure S4). Figure S5 shows the dependence of the capacity values on the content of SWCNT in the cathode composite. The capacity of the electrodes tends to grow with an increase in the SWCNT content from 0 to 0.25 wt.% but remains on a similar level upon further addition of SWCNT (Figure S5). The effect of SWCNT is observed even at a low charge/discharge rate (C/10) and becomes much more pronounced at higher current densities (10C). The higher the charge/discharge rate, the stronger the capacity depends on the nanotube content (Figure S5). Thus, it can be concluded that small variations in LFP or PVDF amounts do not affect the optimal amount of SWCNT (0.25 wt.%) to be added to the electrode to provide electrical conductivity.

Cycling stability is an important performance characteristic of electrodes which affects the lifetime of a Li-ion battery. Figure 5b compares the cycling performance of the electrode containing no SWCNT and those with varying amounts of SWCNT. The electrode with no SWCNT demonstrates fast capacity fading, retaining only 16% of initial capacity by the 100th cycle. However, CE for this electrode is relatively high (~99.7%) and does not decrease with cycling (Figure 5b). Hence, the capacity fading is not related to electrochemical irreversibility or incomplete discharge. At the same time, a minor addition of SWCNT (0.05 wt.%) improves the cycling stability (capacity fading is 27% over 100 cycles). The electrode with 0.25 wt.% of SWCNT has the best cyclability, and its capacity loss is only 2% after 100 cycles. Thus, the content of SWCNT in the cathode material plays a key role in providing the cyclic stability of the cathodes. The network formed by SWCNT enhances both the electrical conductivity and mechanical strength of the composite cathode, thereby significantly impacting its cyclic characteristics [46]. During cycling, mechanical stress causes the loss of electrical contact between adjacent LFP particles and between LFP particles and the current collector, thus resulting in capacity fading. Poor electrical contacts can also deteriorate due to the heat generation effect that is observed at high current densities [24]. SWCNT prevents capacity loss by providing additional conductive pathways within the electrode. The cycling characteristics of the electrodes confirm our conclusion that the most effective content of SWCNT in the composite cathode is 0.25 wt.%.

### 3.3. Effect of the Binder Amount

**Figure 6:**
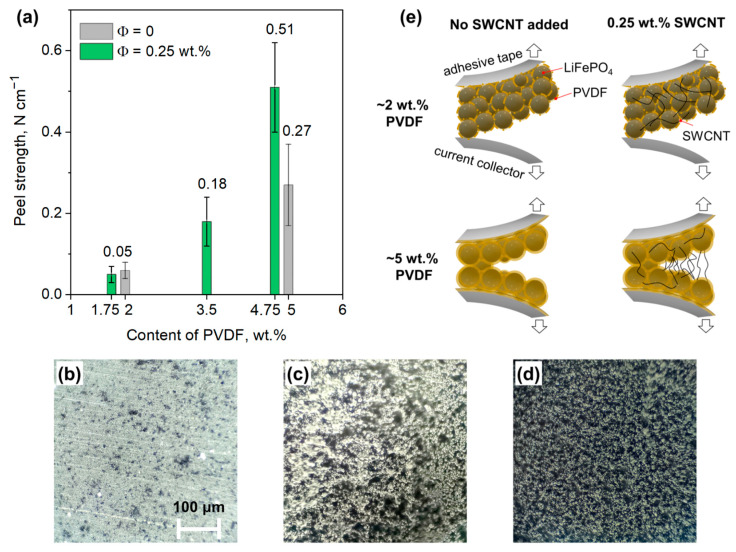
(**a**) Peeling resistance of the LFP cathodes formulated with different contents of PVDF binder; optical micrographs of (**b**) pristine Al-C current collector surface, (**c**,**d**) current collector surfaces after peeling off the cathodes containing 0.25 wt.% SWCNT and different amounts of the binder: (**c**) 1.75 wt.% PVDF, (**d**) 4.75 wt.% PVDF; (**e**) proposed failure mechanisms in peel-off tests depending on the presence of SWCNT and the amount of PVDF binder in cathode composite. The cathodes contained 95 wt.% of sphere-shaped LFP particles.

The rational design of a cathode composition also requires careful optimization of the amount of the polymer binder. This component determines the adhesive properties and mechanical durability of electrode [48] and can affect the cycle life of the battery. Our attempts to introduce very small amounts of PVDF binder (≤1 wt.%) were unsuccessful since the resulting electrode slurries lacked viscosity and uniformity, while the composite electrodes were highly brittle and delaminated spontaneously from the current collector during roll-pressing and punching procedures. The minimal amount of binder allowing for the fabrication of mechanically robust electrodes was ca. 1.75 wt.%. To further optimize the content of PVDF, we prepared a series of LFP cathodes with varying amounts of PVDF (1.75–4.75 wt.%) and a previously optimized amount of SWCNT (Φ = 0.25 wt.%). The mechanical properties of these electrodes were characterized by the standard peel-off test technique. To assess a possible contribution of carbon nanotubes to the mechanical properties of the electrodes, we also investigated the peeling behavior of some electrodes prepared with no SWCNT added (Φ = 0). The measured peel strength values are shown in Figure 6a as a function of PVDF content.

The analysis of the Figure 6a allows us to draw the following conclusions: (1) electrodes comprising a relatively small amount of binder (1.75–2 wt.% of PVDF) demonstrate the peeling resistance of ca. 0.05 N cm^−1^, regardless of the presence of carbon nanotubes in the electrode; and (2) increasing the content of PVDF from 1.75 to 4.75 wt.% improves the mechanical strength of the electrode by 10 times. These results correspond well with the literature data that indicates a strong correlation between the amount of binder and the peeling resistance of the electrode [49]. Greater amounts of binder ensure better coverage of the current collector, active particles, and electrically conducting component, thus resulting in better mechanical coupling between them. Surprisingly, the electrode containing 0.25 wt.% of SWCNT and 4.75 wt.% demonstrates two times higher peeling strength as compared to the electrode prepared with no SWCNT and a comparable amount of PVDF (5 wt.%, Figure 6a).

Hence, SWCNT contributes to the overall peeling force of the electrode. To explain this fact, we analyzed electrode mechanical failure mechanisms using optical microscopy [25,49,50]. In each case, the peeled-off adhesive tapes contained a dark-colored layer of electrode material, while the peeled-off current collectors looked different depending on the electrode composition. The image of a pristine Al-C current collector includes light areas, which correspond to the bare Al/Al_2_O_3_ foil surface, along with darker ~5 µm areas, which represent carbon particles (Figure 6b). The electrodes formulated with a small amount of PVDF binder (1.75 wt.%) delaminate from the Al-C foil almost entirely, exposing large light areas of aluminum surface (Figure 6c). It implies the mostly adhesive type of electrode failure [49,50]. In this case, the measured peeling force is mostly adhesive, and it does not depend on the presence of a small amount of SWCNT in the electrode (Figure 6e). The electrodes that are prepared with higher PVDF content (4.75 wt.%) remain both on the current collector and the peeling tape after the peel-off test. The delaminated current collector is dark colored, and the metallic surface is hardly seen (Figure 6d), which indicates a mostly cohesive type of mechanical failure [49,50]. In this case, a high amount of PVDF binder efficiently prevents the delamination of the electrode from the surface of the current collector, so that failure occurs inside the electrode rather than on the electrode/current collector interface. The addition of SWCNT improves the contact between the separated particles of the binder (Figure 1h) and enhances the cohesive strength of the electrode (Figure 6e).

The cycling performance of the electrodes containing different PVDF amounts is presented in Figure S6. The electrodes prepared with different amounts of the binder yield nearly equal capacities. Reducing the content of the binder from 4.75 to 1.75 wt.% does not affect the cycling stability over the 100 charge-discharge cycles. It is important to note that a smaller amount of polymer binder increases the content of active material and, consequently, enhances the packing density and volumetric and areal capacity of the cathode composite as well as reduces its production cost. Therefore, the most effective amount of binder is about 2 wt.%, which provides the best balance between the mechanical and capacitive characteristics of the cathodes. However, more PVDF binder (~5 wt.%) may be required for more prolonged cycling of the battery since it efficiently prevents a possible adhesive failure of the cathode.

### 3.4. Effect of the Electrode Thickness

The thickness of the cathode layer is an important parameter that affects the energy and power densities of a Li-ion battery. Increasing the thickness of the electrode efficiently improves the energy density value by reducing the relative fraction of electrochemically inactive materials (separator and current collector) [51]. To evaluate how the thickness of the electrode affects its performance, we used previously optimized cathode slurry composition (98 wt.% LFP, 1.75 wt.% SWCNT, and 0.25 wt.%) to form thicker cathode layers with areal loadings of LFP up to 45 mg cm^−2^. The prepared electrodes remained mechanically robust even after the current collector was delaminated.

**Figure 7:**
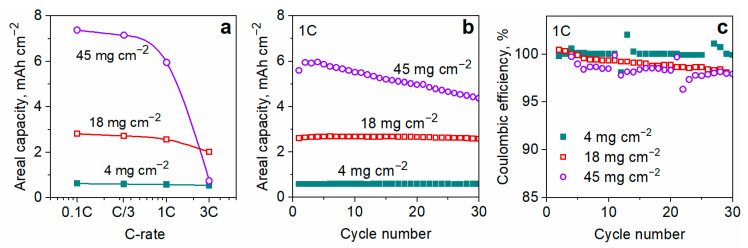
(**a**) Rate performance, (**b**) 1C-rate cycling stability, and (**c**) coulombic efficiency of the cathodes with different loadings of spherical LFP particles. The cathodes contained 98% LFP, 1.75% PVDF, and 0.25% SWCNT. The cathode with 4.0 mg cm^−2^ loading of LFP was prepared on an Al-C current collector while the thicker ones were free-standing. The capacity values are normalized by the cathode surface area.

Rate capability is a crucial characteristic of the high mass loading electrodes because they can usually operate only at low current densities (C/10–C/5) [52]. Figure 7a compares the area-normalized capacities of a pristine electrode (4.0 mg cm^−2^ LFP, Al-C current collector) and thicker free-standing electrodes (18 and 45 mg cm^−2^ LFP) at different charge/discharge rates.

At a low charge/discharge rate (0.1C), the specific capacities of the tested electrodes are similar (157–159 mAh g^−1^). The areal capacities are proportional to the corresponding LFP loadings and reached 2.8 and 7.4 mAh cm^−2^ (C/10) for the electrodes with 18 and 45 mg cm^−2^ LFP, respectively (Figure 7a). The electrode with the lowest LFP loading (4 mg cm^−2^) exhibits excellent capacity retention at high current densities (87% retention after increasing the rate up to 3C). The electrode with 18 mg cm^−2^ of LFP retains 72% of its initial capacity at 3C, whereas a thicker electrode (45 mg cm^−2^ LFP) fails to perform at rates > 1C. The rate performance of the thicker electrodes is limited by the Li-ion diffusion rate, as reported earlier [51,53]. Accordingly, the electrode with an intermediate loading of LFP (18 mg cm^−2^ LFP) demonstrates a much higher areal capacity at 3C (2.0 mAh cm^−2^) compared to electrodes with lower or higher LFP loadings (Figure 7a). The thickness of the electrode affects both ohmic resistance and lithium-ion transfer rate. It is known that electronic resistance is proportional to the thickness of the electrode. Thus, the increase of the thickness contributes to the rise in electronic resistance of the electrode resulting in the large overvoltage during charge-discharge. The increase in the Li-ion diffusion distance (often called “characteristic diffusion length”) in the liquid phase within the pores in thicker electrode results in mass transfer limitations. These two factors strongly distort the voltage-composition profile, leading to a sharp increase or decrease in potential, as a result of which a voltage cutoff is triggered, and the system cannot gain its full capacity at elevated charge/discharge rates. Moreover, it is known that active LiFePO_4_ particles undergo volume expansion and contraction during lithium insertion and de-insertion processes. The volume change induces stress accumulation within the thick electrode which is hard to release and can cause cracking or fracture of the electrode. Cracks that appear within the electrode cause the particle isolation leading to areal capacity loss.

The cyclability of the electrodes with varied LFP loadings is shown in Figure 7b. The electrodes with 4 and 18 mg cm^−2^ LFP loadings demonstrate stable cycling behavior at 1C, while the capacity of the thicker electrode (45 mg cm^−2^ LFP) decreases by 22% over 30 cycles. The capacity fading can be attributed to the mechanical degradation and side chemical reactions occurring in the highly polarized thick electrode [52] as well as to a fast lithium dendrite growth due to a large amount of Li^+^ that participates in the electrochemical reaction. As can be seen from Figure 7, the cell with high-loading cathode exhibits the highest degradation during cycling and the lowest CE. We believe that these two factors (fast degradation and low CE) are interrelated and that both of them refer to the anode part of the cells. The cathode with 45 mg/cm^2^ of LFP contains a much larger absolute amount of lithium, which is deposited on the anode at each cycle and dissolves again to pass back into the cathode. It is worth noting that the area of the counter electrodes and the amount of electrolyte in the cells with different cathode loadings are the same. It is well known that lithium is deposited unevenly on the anode surface, and the solid-electrolyte interface (SEI) is constantly updated. This, in turn, is accompanied by constant electrolyte consumption and reduced CE values.

Hence, it can be concluded that the free-standing cathode with 18 mg cm^−2^ loading of LFP provides the best balance between the areal capacity, rate performance, and cycling stability. The absence of an electrochemically inert current collector contributes greatly to the overall energy density of this electrode. Moreover, the gravimetric capacity of the thin electrode (4 mg cm^−2^ loading of LFP) formulated on an Al-C current collector is only 43 mAh g^−1^ (C/10) when normalized to the total electrode weight that includes Al foil, LFP material, PVDF binder, and the SWCNT additive. The similar characteristic is 3.6 times higher for the free-standing 18 mg cm^−2^ LFP electrode (156 mAh g^−1^), which is close to the theoretical capacity of LFP (170 mAh g^−1^).

## 4. Discussion

We compared the electrochemical performance of two promising morphological types of LFP material, namely, LFP nanoplates and LFP microspheres. The spherical LFP provides higher volumetric capacity (Table 1), but poorer contact with an aluminum current collector, which results in inferior rate capability (Figure 2a). This issue can be effectively resolved by coating the current collector with a thin layer of carbon, which promotes good interfacial contact (Figure 3c) and improves rate capability (Figure 2b).

Then, we investigated how the amount of SWCNT affects the electrochemical performance of an electrode. The electrode slurries were prepared without using SWCNT dispersion stabilizers, which makes the developed procedure economically feasible. SWCNT formed elongated bundles within the electrodes, which promoted the formation of stable percolation network. Increasing the SWCNT content up to 0.25 wt.% results in a significant decrease in charge transfer resistance and improves electrical conductivity, rate capability, and cycling stability of the electrodes (Figure 4 and Figure 5) with no further enhancement at higher SWCNT loadings.

The peeling force of the electrodes is strongly influenced by the amounts of both PVDF binder and SWCNT additive. The amount of PVDF can be reduced to only 1.75 wt.%; the resulting electrodes with ultra-high active material content (98 wt.%) demonstrate excellent cycling stability (the capacity fading is less than 2% over 100 cycles, Figure S6). Increasing the amount of PVDF from 2 to 5 wt.% changes the type of electrode mechanical failure from adhesive to cohesive (Figure 6). SWCNT does not affect the adhesive strength of the electrode but helps improve its cohesive strength and peeling resistance by two times.

The determined optimal composition of the electrode is further used to formulate free-standing high mass loading LFP cathodes. SWCNT provides mechanical durability of the cathodes without a current collector. The cathode with 18 mg cm^−2^ areal loading of LFP offers the best balance between the energy and power performance (Figure 7a), having a high areal capacity (2.8 mAh cm^−2^ at C/10 and 2.0 mAh cm^−2^ at 3C) and a gravimetric capacity close to theoretical. The areal capacity of this electrode is comparable to that of commercial high-energy-density oxide cathodes based on LiNi_1/3_Co_1/3_Mn_1/3_O_2_ [51].

Table 2 compares the performance of the developed electrodes with previously reported ones.

The electrodes with low loading of LFP (4–7 mg cm^−2^) can operate at a high charge/discharge rate of 10C. The electrode reported in our study demonstrates a comparable 10C-rate capacity (100 mAh g^−1^) and still incorporates a lower amount of CNT and a higher amount of LFP than typical literature electrodes (Table 2). High-energy-density LFP-based electrodes (~40 mg cm^−2^ LFP) are also compared in Table 2. Our electrode demonstrates a higher 1C-rate areal capacity (5.95 mAh cm^−2^) as compared to the literature data.

## 5. Conclusions

In this work, we optimized the composition of LFP-PVDF-SWCNT cathodes to meet the requirements of high-power-density applications. The spherical morphology of LFP particles was favorable for achieving high packing density. The interfacial contact between the current collector and LFP particles was a key factor affecting the rate capability of a cathode. The use of a carbon-coated current collector reduced the interfacial resistance which resulted in an improved cathode volumetric capacity (255.0 mAh cm^−3^ at 1C).

A minor addition of SWCNT enhanced electrical conductivity, rate capability, cyclic performance, cohesion strength, and peeling resistance of the electrodes. The optimal content of SWCNT in the electrode was determined to be 0.25 wt.%, which allowed ultra-high active material content (98 wt.% of LFP). The electrodes with SWCNT can form thick free-standing electrodes with a high areal capacity (up to 5.9 mAh cm^−2^ at 1C).

Taking into account the advantages of LFP material including good safety, low cost, and constant discharge potential, we consider the developed LFP cathodes promising for application in electric vehicles and other high-energy-density devices.

## Figures and Tables

**Table 1:** Volumetric characteristics of the composite cathodes prepared with LFP particles of different morphologies ^1^.

Morphology of LFP Particles	Tap Density of LFP Particles, g cm^−3^	Electrode Density, g cm^−3^	Volumetric Capacity of Electrode ^2^, mAh cm^−3^
plates	0.8	0.81 ± 0.2	116.1
spheres	1.7	1.76 ± 0.2	255.0

^1^ The electrodes incorporated 95 wt.% of LFP, 4.75 wt.% of PVDF, and 0.25 wt.% of SWCNT. Al-C was used as a current collector. ^2^ at 1C charge/discharge rates

**Table 2:** The characteristics of cathodes formulated with LFP and SWCNT. (n/a—not available).

Content of LFP, wt.%	Loading of LFP, mg cm^−2^	Content of CNT, wt,%	Density of Electrode, g cm^−3^	Specific Capacity (10C-Rate), mAh g^−1^	Areal Capacity (1C-Rate), mAh cm^−2^	Free-Standing Electrode	Reference
95	4.0	0.25	1.8	100	0.39	–	This study
95	4.8	0.25	1.4	105	0.50	–	[25]
95	7.0	5	n/a	105	0.96	+	[54]
81	6.0	4	n/a	118	0.79	+	[54]
86	n/a	4.6	n/a	100	n/a	–	[55]
98	45	0.25	1.8	n/a	5.95	+	This study
90	40	1	1.3	n/a	5.1	–	[18]

## Data Availability

Not available.

## Supplementary Materials

The following supporting information can be downloaded at: https://www.mdpi.com/article/10.3390/nano13111771/s1, Figure S1: Typical first-order Raman spectrum of the surface of an Al-C current collector; Figure S2: Galvanostatic charge and discharge profiles (1C-rate) for the composite electrodes prepared with different types of LFP particles and different current collectors; Figure S3: Galvanostatic charge and discharge profiles of the first three cycles of the composite electrodes with different SWCNT contents; Figure S4: Rate performance of the composite electrodes containing different amounts of LFP (sphere-shaped), PVDF, and SWCNT; Figure S5: Effect of SWCNT content on the capacity of LFP-based composite electrodes; Figure S6: Cycling performance of the electrodes with different contents of PVDF binder.
