# Supplementary material for: Engendering High Energy Density LiFePO4 Electrodes with Morphological and Compositional Tuning
Source: Nanomaterials (Basel). 2023 May 31;13(11):1771. doi: 10.3390/nano13111771 (PMC10254251; doi:10.3390/nano13111771)
Supplement: Supplementary file 1 [file nanomaterials-13-01771-s001.zip › nanomaterials-2372306-supplementary.pdf]

Supplementary Materials

Engendering High Energy Density $LiFePO_4$ Electrodes with Morphological and Compositional Tuning

Aleksei V. Kubarkov,[a,*] Alexander V. Babkin,[a] Oleg A. Drozhzhin,[a,b] Keith J. Stevenson,[a] Evgeny V. Antipov,[a,b] and Vladimir G. Sergeyev[a,*]

[a] Department of Chemistry, Lomonosov Moscow State University, Leninskie Gory 1-3, 119991 Moscow, Russia;

[b] Center for Energy Science and Technology, Skolkovo Institute of Science and Technology, Bolshoy Boulevard 30 bld. 1, 121205 Moscow, Russia

* Corresponding authors: aleksei.kubarkov@gmail.com, sergeyev@genebee.msu.ru

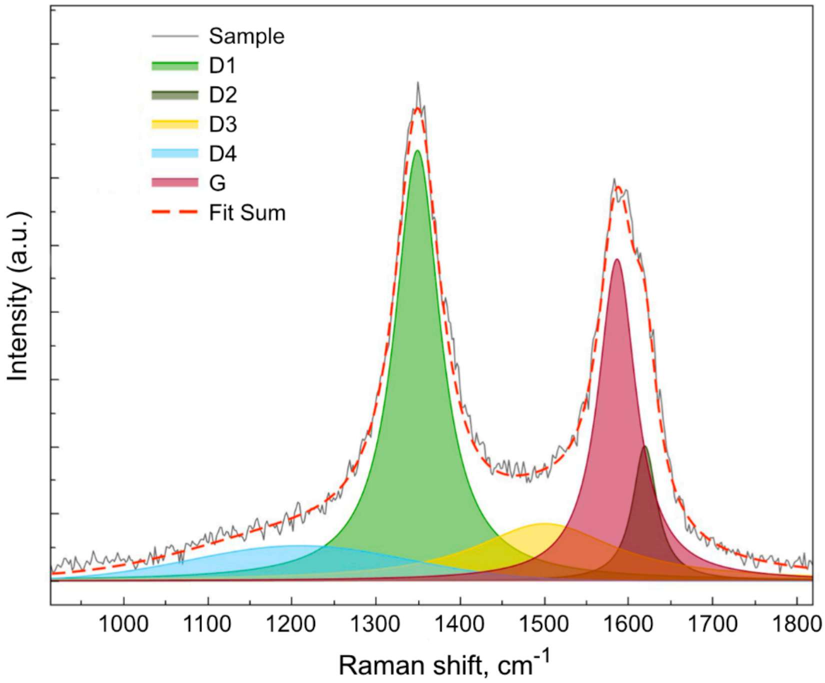

Figure S1. Typical first-order Raman spectrum of the surface of an Al-C current collector. The spectrum was fitted with four Lorentzian-shape bands (D1, D2, D4 and G) and a Gaussian-shape band (D3).

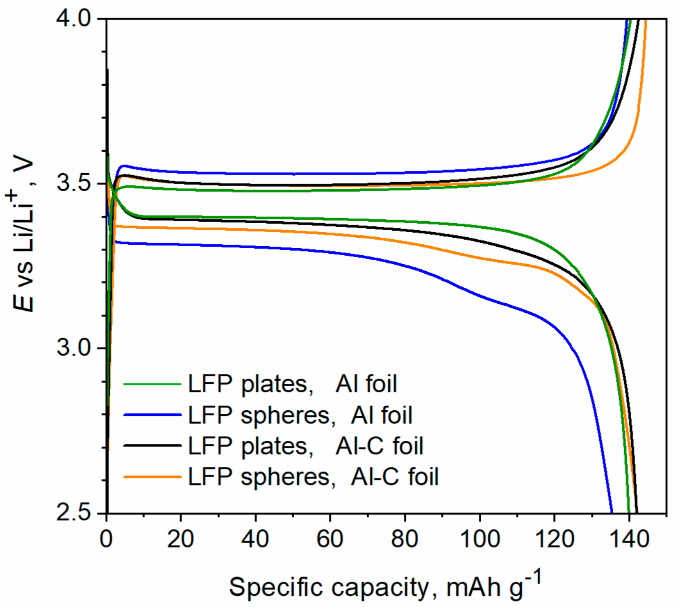

Figure S2. Galvanostatic charge and discharge profiles (1C-rate) for the composite electrodes prepared with different types of LFP particles and different current collectors.

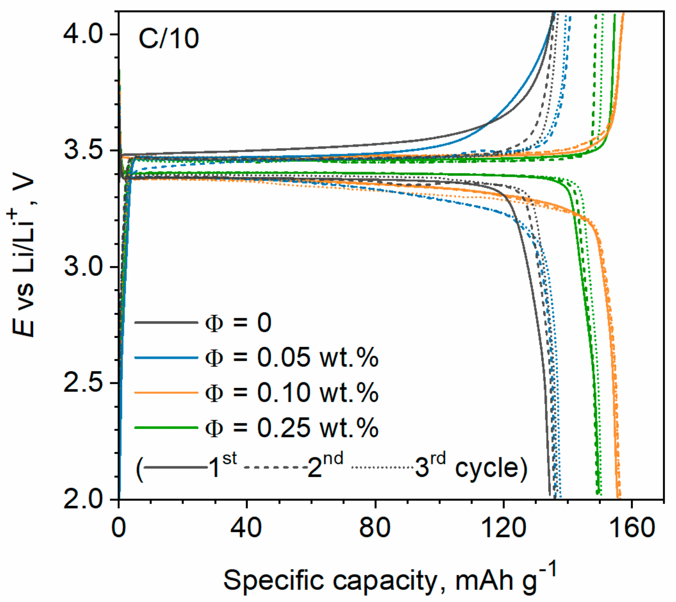

Figure S3. Galvanostatic charge and discharge profiles of the first three cycles of the composite electrodes with different SWCNT contents (Φ).

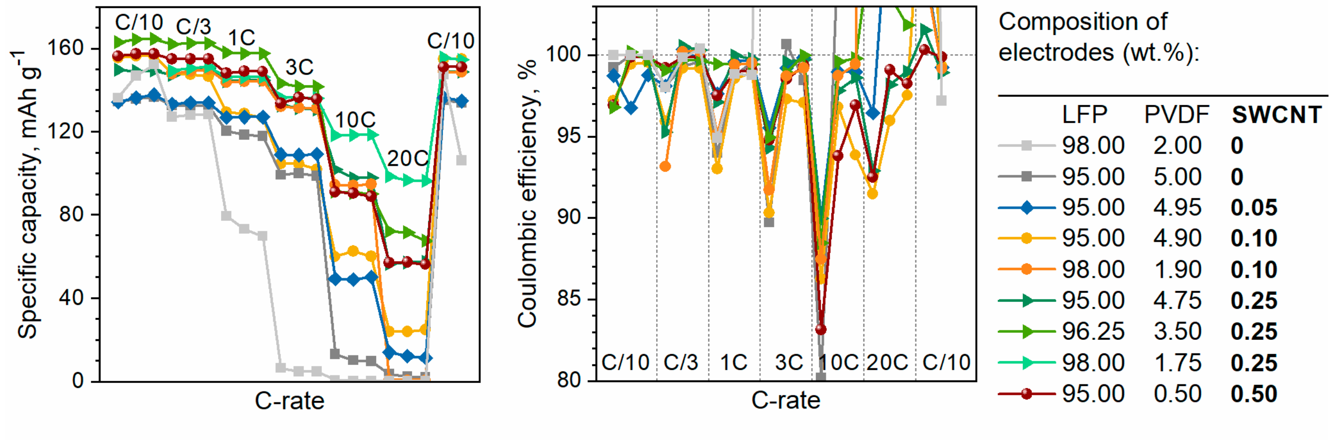

Figure S4. Rate performance of the composite electrodes containing different amounts of LFP (sphere-shaped), PVDF, and SWCNT.

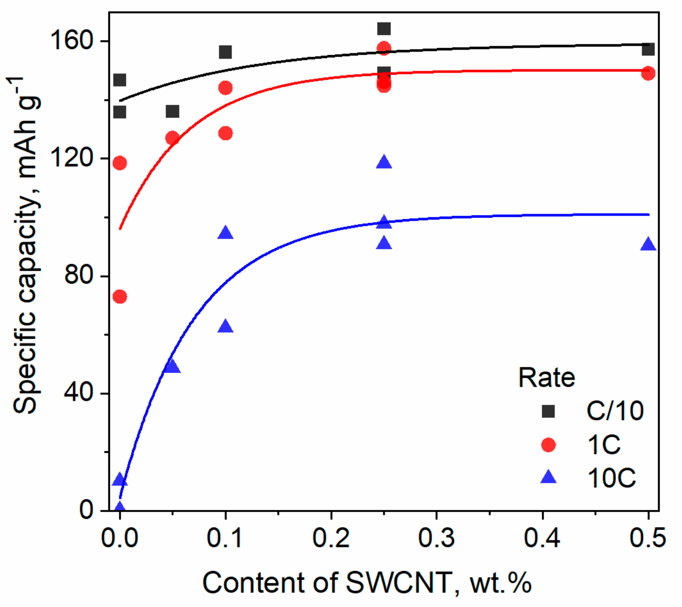

Figure S5. Effect of SWCNT content on the capacity of LFP-based composite electrodes. The composition of the studied electrodes is shown in Figure S4.

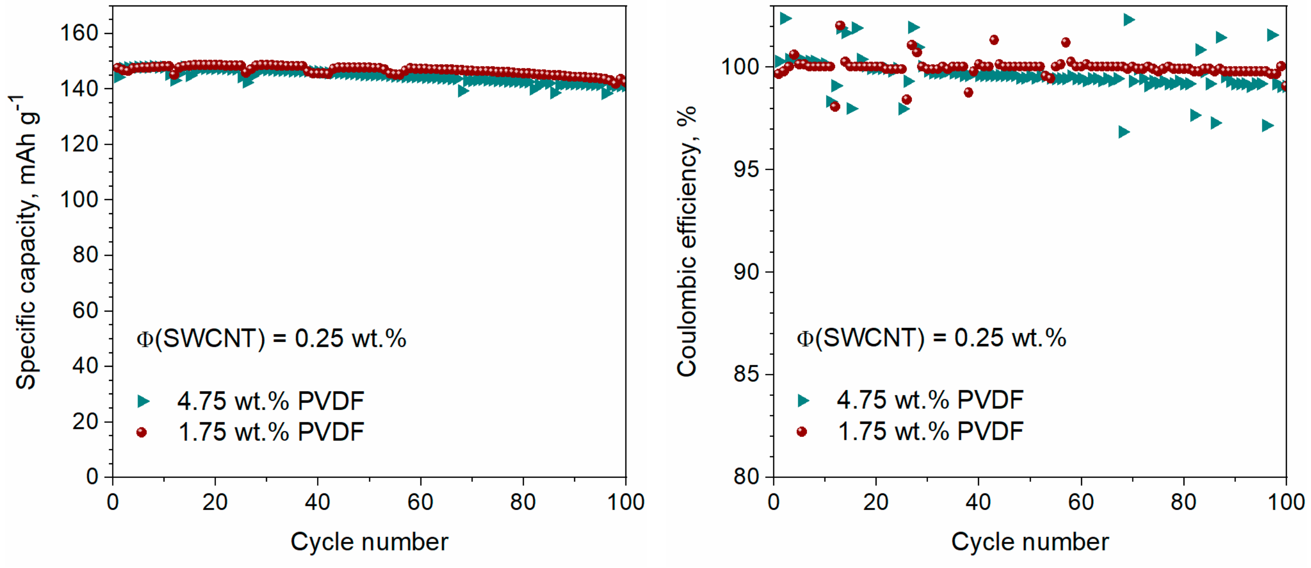

Figure S6. Cycling performance of the electrodes with different contents of PVDF binder.
